# Hepatitis C Virus entry: the early steps in the viral replication cycle

**DOI:** 10.1186/1743-422X-6-117

**Published:** 2009-07-30

**Authors:** Ali Sabahi

**Affiliations:** 1 Department of Microbiology and Immunology, Tulane University Health Sciences Center, New Orleans, Louisiana, USA

## Abstract

Approximately 170 million are infected with the hepatitis C virus (HCV) world wide and an estimated 2.7 million are HCV RNA positive in the United States alone. The acute phase of the HCV infection, in majority of individuals, is asymptomatic. A large percentage of those infected with HCV are unable to clear the virus and become chronically infected. The study of the HCV replication cycle was hampered due to difficulties in growing and propagating the virus in an *in vitro *setting. The advent of the HCV pseudo particle (HCVpp) and HCV cell culture (HCVcc) systems have made possible the study of the HCV replication cycle, *in vitro*. Studies utilizing the HCVpp and HCVcc systems have increased our insight into the early steps of the viral replication cycle of HCV, such as the identification of cellular co-receptors for binding and entry. The aim of this article is to provide a review of the outstanding literature on HCV entry, specifically looking at cellular co-receptors involved and putting the data in the context of the systems used (purified viral envelope proteins, HCVpp system, HCVcc system and/or patient sera) and to also give a brief description of the cellular co-receptors themselves.

## Introduction

### Epidemiology

Approximately 170 million are infected with the hepatitis C virus (HCV) world wide. HCV is a positive strand RNA virus belonging to the *flaviviridae *family and is the sole member of the genus *Hepacivirus*. It is a hepatotropic virus which replicates in the cytoplasm of hepatocytes. In the United States an estimated 2.7 million are HCV RNA positive [1]. Most individuals infected with HCV show little or no symptoms during the acute phase of the infection. Of those infected with HCV, 54–86% fail to clear the virus and develop a chronic infection. The chronic phase can last many decades and can ultimately lead to end stage liver disease. In retrospective studies in individuals with chronic HCV infections, cirrhosis of the liver occurred in 17–55%, hepatocellular carcinoma (HCC) developed in 1–23%, and liver related death occurred in 4–15%. In prospective studies, cirrhosis developed in 7–16% of chronically infected individuals, HCC occurred in 0.7%–16%, and liver related death in 1.3–3.7% [2]. HCC, by itself, is the third leading cause of cancer related deaths worldwide with 40.1% of patients with HCC being anti-HCV positive [3].

### Treatment options and efficiency

Since the initial acute phase of a HCV infection is in most cases asymptomatic, most infected individuals seeking treatment are chronically infected. The goal of any treatment is to achieve a sustained virological response (SVR), which is the absence of serum HCV RNA up to 6 months after therapy is concluded. The initial tool for treatment for a HCV infection was mono-therapy with interferon-α (IFN-α). An improvement was made to this therapy with the introduction of pegylated interferon-α (peg-IFN-α). The purpose and result of the pegylation of IFN-α was an increase in the half life of the drug, *in vivo*, from a few hours to days. This resulted in an increase of greater than 100% in achievement of SVR when compared to treatment with IFN-α [4]. To increase efficiency of the treatment, peg-IFN-α therapy has been supplemented with ribavirin. Combination therapy with peg-IFN-α and ribavirin has resulted in a further increase in treatment efficiency with 54% of HCV infected patients achieving SVR.

The response and rate of SVR is dependent on the genotype of HCV with only 30% of genotype 1 infected individuals achieving SVR, whereas greater than 80% of genotype 2 or 3 achieve SVR with combination therapy [4]. Combination therapy treatment regiments are genotype dependent and the amount of peg-IFN-α administered is dependent on the type used. For peg-IFN-α-2a a dose of 180 μg/week is prescribed during the course of the therapy. For peg-IFN-α-2b a dose of 1.5 μg/kg/week is prescribed. For those infected with HCV genotypes 1, 4, 5, or 6, peg-IFN-α is prescribed in combination with 1000 mg/day (75 kg or less) or 1200 mg/day (greater than 75 kg) of ribavirin. For those infected with genotype 2 or 3 the duration of treatment is 24 weeks with the combination of peg-IFN-α and 800 mg/day of ribavirin prescribed [5].

Of those that do not respond to therapy, and continue to be chronically infected, a percentage will develop HCC or decompensation and therefore require a liver transplant. For those with an active HCV infection, reinfection after transplantation is universal. Reinfection occurs during liver reperfusion with HCV levels reaching pre-transplant levels in a period of 72 hours. Post-transplantation, the steady state of HCV viral load is 10 times higher than pre-transplantation. Of those that develop post-transplantation cirrhosis, 42% develop decompensation and only 50% survive one year after the development of decompensation. Living donor liver transplant (LDLT) allows for the pre-treatment of patients, prior to the transplantation, to lower the viral load or eradicate the virus. This leads to a very low (10%) post-transplantation viral reoccurrence [6].

### Genomics and Proteomics

The hepatitis C virus (HCV), a positive stranded RNA virus, is the sole member of the *Hepacivirus *genus within the *Flaviviridae *family. The HCV genome is 9.6 kb with a 5' NCR, followed by an open reading frame coding for structural and non-structural proteins, and 3' NCR region. Within the 5' NCR region resides an internal ribosome entry site (IRES) which drives the translation of the genome. The product of the translation process is a 3000 amino acid long polyprotein. The polyprotein is cleaved by viral and cellular enzymes (signal peptidases) to individual proteins. The structural proteins are the core protein and the envelope glycoproteins, E1 and E2. The non-structural proteins are the P7 ion channel, the NS2-3 protease, the NS3 serine protease and RNA helicase, the NS4A polypeptide, the NS4B and NS5A proteins, and the NS5B RNA-dependent RNA polymerase (RdRp) [7].

The NS5B RdRp lacks proof reading function, and coupled with the high rate of replication of the virus, leads to the production of a viral pool with high level of genetic variability. HCV isolates are classified into genotypes and subtypes [8]. There are 6 major genotypes that differ in nucleotide sequence by 30–50% and several subtypes within a genotype that differ in nucleotide sequence by 20–25%. The term quasispecies refers to the genetic heterogeneity of the viral pool found in an infected individual [8]. Of the six different genotypes, genotype 1 is the most resistant to current therapy for HCV infection.

### In vitro models of HCV infection

Since the discovery of HCV different *in vitro *models have been used to study the viral replication cycle. The first *in vitro *system of significance was the HCV replicon system. In a prototype HCV replicon the HCV IRES drives the translation of a neomycin phosphotransferase gene followed by a heterologous (ECMV) IRES driving the translation of the HCV structural and nonstructural (full length replicon), or nonstructural genes (subgenomic replicon) [9]. The HCV replicon system allowed for the first time the study of HCV RNA replication but not the whole viral replication cycle. Cells transfected with the HCV replicon, although replicating HCV RNA at high levels, were incapable of producing infectious virus. An *in vivo *study in chimpanzees supported the hypothesis that the adaptive mutations required for efficient replication of the HCV genome *in vitro *interfered with virus packaging and secretion [10].

The HCV replicon system allowed for the study of HCV RNA replication. To understand the process of entry a HCV pseudo-particle (HCVpp) system was contrived [11,12]. HCVpp is made by transfecting 293T cells with 2 plasmids, one containing an envelope deficient HIV proviral gene, with a luciferase cassette, and the second containing the HCV glycoproteins. The particles produced can then be used to infect naive cells and the level of infectivity can be measured by a luciferase assay. The HCVpp system allowed for the study of early infection events, binding and entry, of the HCV replication cycle.

In 2003 a HCV genotype 2a clone was isolated from a Japanese patient with a rare case of fulminant hepatitis C. This clone was designated as JFH1 (for Japanese fulminant hepatitis 1) and the replicon constructed from this strain was found to replicate in Huh-7 cells (hepatoma cell line) without the need for adaptive mutations [13]. Subsequently, it was found that transfection of JFH1 RNA into Huh7 cells resulted in the de novo production of infectious virus (designated HCVcc for cell culture derived HCV) that is capable of infecting naive Huh7 cells [14-16]. The virus produced in tissue culture was infectious in chimpanzees [14,17] and in immunodeficient mice with partial human livers, and the virus inocula derived from these animals was infectious for naive Huh7 cells [17].

### HCV replication cycle

HCV infection is a highly dynamic process with a viral half life of a few hours and production/clearance of an estimated 10^12 ^virions per day in an infected individual [18]. Upon binding to hepatocytes, HCV enters cells by clathrin-mediated endocytosis [19]. A number of cellular co-receptors of HCV have been identified. They include glycosaminoglycans [20-24], the LDL receptor (LDLR) [25,26], DC-SIGN and L-SIGN [27-29], CD81 [24,30-47], SRBI [48-56], and claudin-1 [57-59]. Current evidence suggests that within the endosome, the low pH environment triggers the fusion process of the virus with the endosomal membrane and the introduction of the HCV genome into the cytoplasm [60-62].

Translation of the HCV genome is driven by the IRES located in the highly conserved 5' NCR. Initiation of translation occurs through the formation of a complex of the HCV IRES and the 40S ribosomal subunit. This event is followed by association eIF3 and the ternary complex of eIF2Met-tRNAGTP and the formation of a 48S-like complex at the initiation codon of the HCV RNA. The final and rate limiting step is the GTP-dependent association of the 60S subunit to form the 80S complex [63]. The translation process and subsequent processing by viral and cellular proteases yields mature structural and non-structural proteins. The structural proteins and p7 polypeptide are processed by the endoplasmic reticulum (ER) signal peptidase and the nonstructural protein are processed by the NS2-3 protease and NS3-4A serine protease [7].

The expression of the HCV proteins leads to the formation of replication complexes in the cytosol. The replication complexes are situated near the cell membrane which can be visualized as a membrane alteration called the membranous web [64,65]. It has been recently shown that the binding of a liver specific micro-RNA (miRNA), miRNA122, to the 5' NCR of HCV enhances the viral RNA replication process [66]. The expression of HCV proteins and the replication of the HCV genome is followed by the packaging of the virus particles and secretion. Presumably virions form by budding into the ER and exiting through the secretory pathway.

### HCV association with lipoproteins and particle density

Current evidence indicates that HCV particles, both *in vitro *and *in vivo*, exist as virus-lipoprotein particles with a broad density profile [15,67-70]. The density profile of a HCV positive serum sample from a chronically infected patient displayed a distribution from 1.13–1.04 g/ml, with the majority of the HCV RNA being at 1.08 g/ml and below. At pH 4 the density shifted slightly toward higher densities and an increase to pH 9.2 had no effect on the density profile. Immunoprecipitation experiments using ApoB and ApoE antibody showed that at densities below 1.06 g/ml the HCV particles from the serum sample were associated with ApoB and ApoE, which suggests association of these viral particles with LDL and VLDL. This association decreased as particle densities increased [68].

The density profile of HCVcc particles shows an HCV RNA distribution from 1.0 to 1.18 g/ml with a peak at 1.13 to 1.14 g/ml. The HCVcc infectivity profile displays a broad distribution from 1.01 to 1.12 g/ml with no infectivity at densities greater the 1.12 g/ml [15]. The HCV RNA and infectivity peaks of the density profile HCVcc do not overlap and there is little or no infectivity at the density of the RNA peak. This fraction has been shown to largely contains a RNase resistant encapsidated HCV RNA particles which are non-infectious [67].

## HCV entry cellular receptors

### CD81

CD81 was recognized early as an entry receptor for HCV [43]. CD81 is a member of the tetraspanin family of proteins. Tetraspanins are type III membrane glycoproteins which span the membrane 4 times and therefore producing 2 extracellular loops and a short intracellular loop. Of the 2 extracellular loop, the long extracellular loop (LEL) contains the signature structural feature of the tetraspanin family of proteins. There are disulfide bonds between the 4 cysteine residues in the LEL which form a subloop structure containing a region that is hypervariable between family members. The region outside the subloop contains greater structural conservation among family members, forming 3 alpha helices. Tetraspanins have no intrinsic enzymatic activity. They form structures on the plasma membrane called tetraspanin enriched microdomains (TEMS) which are distinct from lipid rafts although they have been shown to interact physically. Although there has been evidence that tetraspanins interact with counter receptors on other cells, most evidence indicates that they instead act in cis with other transmembrane proteins and regulate post-ligand binding events, including integrin-mediated adhesion strengthening. The c-terminus of CD81, CD151, and other tetraspanins meet the criteria for being recognized by either type III or type I PDZ domains therefore leaving open the possibility of interaction with the cytoskeleton. Previous studies have shown that tetraspanins affect such processes such as cell proliferation, apoptosis, and tumor metastasis [71,72].

Due to the lack of an *in vitro *infectious system, early studies utilized soluble E2 (sE2, lacking the transmembrane region) to identify CD81 as a HCV receptor [31,36,43,73-76]. The binding strength, K_d_, of sE2 to CD81 LEL were experimentally found to be at 1.8 nM at 25°C and 9.1 nM at 37°C, and the formation of disulfide bonds among the 4 cysteines in the LEL are necessary condition for sE2 binding to the CD81 LEL [77]. The role of CD81 as a cellular receptor for HCV was further strengthened with the advent of the HCVpp [32,34,37,40,41,47,49,55,78] and HCVcc [24,30,38,55] systems. It has been demonstrated that HCV and HCV glycoprotein E2 bind CD81 and not other members of the tetraspanin family [36]. Binding of E2 occurs at the CD81 LEL and binding of E2 to CD81, or infections with HCVpp or HCVcc, are inhibited with pretreatment with CD81 LEL or antibody versus CD81 [34,36,47,76,77].

Expression of CD81 is not indicative of permissiveness to HCV infection and the expression of human CD81 in cells that are CD81 negative or in cells of other species does not confer susceptibility to HCV infection, with the exception of human CD81 expression in CD81 negative human hepatic cell lines (i.e. HepG2 cell line) [32-34,37,41,47,79]. The level of CD81 expression does not foretell the level of permissiveness to HCV infection. HepG2 cells, a CD81 negative human hepatoma cell line, transfected and expressing CD81 were less susceptible to HCVpp infection than Huh7 cells, a CD81 positive human hepatoma cell line, although expressing higher levels of surface CD81 [34].

The identification CD81 LEL as the domain which interacts with HCV E2 led to studies to discern the E2 binding site on the LEL. It was previously shown that CD81 is normally found as a homodimer on the plasma membrane, and binding studies showed that sE2 binds optimally to a LEL dimer and with much less affinity to a LEL monomer. Furthermore, mutational studies on the LEL identified L162, I182, N184, and F186 as residues that might form part of the E2 binding site. Mutations to these residues do not disrupt the formation of CD81 multimers or the formation of disulfide bonds within the LEL [76].

Antibodies against CD81 were shown to block HCVcc infection if introduced prior to or after the binding of virus to Huh7-Lunet cells at 4°C. In a follow up experiment, cells were infected at various duration, at 37°C, in the presence or absence of anti-CD81, for 10, 20, 30 or 60 minutes. Subsequently, cells were washed and medium was added containing anti-CD81 for 4 hours. The cells were washed and fresh media, without anti-CD81, was added and the efficiency of HCVcc infection was compared to a control infection. Anti-CD81 was able to potently inhibit HCVcc infection, by 60%, even when following an extended binding phase at 37°C, suggesting that CD81 acts at a stage after virus binding [62].

### SRBI

The class B scavenger receptor (SRBI) protein was initially identified as a high affinity low density lipoprotein (LDL) and modified LDL receptor [80,81]. It is a 82KD protein, located primarily to the caveolae, with 2 transmembrane regions, 2 cytoplasmic domains, and large extracellular loop containing a cysteine rich region and 9 putative sites for N-linked glycosylation [82-84]. Its primary function is as a high density lipoprotein (HDL) receptor and its role in cholesterol transport was clarified shortly thereafter [85-88]. SRBI is highly expressed in the liver and steroidgenic tissues, such as the adrenal gland and the ovaries [85,86,89].

Central to the physiological role of SRBI is its primary ligand, HDL. HDL can accept free cholesterol and converts it to cholesterol ester (CE) by a HDL associated enzyme lecithin cholesterol acyltransferase. HDL associated CE can be transferred to other lipoproteins for subsequent transport and metabolism. HDL can deliver the CE to the liver, or steroidgenic tissues in which the CE is used for the production of steroid hormones. In the liver, the HDL-derived cholesterol can be secreted into bile, converted to bile acids, or repackaged into lipoproteins and secreted [89].

The role of SRBI in cholesterol regulation is one of uptake and efflux. The process of selective uptake of free cholesterol (FC) and CE from HDL and LDL particles is largely accomplished without the breakdown of the lipoparticles [82-85,87,88,90-92]. The reverse process of efflux is the movement of cholesterol from the cell to HDL and LDL particles via SRBI [84,88,93,94]. The process of uptake and efflux of cholesterol is inhibited by antibodies against SRBI, which inhibit the binding of lipoprotein, and without an acceptor, such as HDL, there is not an observable transfer of cholesterol from SRBI expressing cells to the extracellular space [94,95].

The importance of SRBI to cholesterol metabolism is further highlighted by work done with mice. In one study targeted mutation of the SRBI genes in mice lead to an increase in plasma cholesterol levels of 30–40% in heterozygote (single knockout mutation) animals and an increase of 2.2 folds in the homozygote (double knockout mutation) as compared to wild type [96]. In a separate study, with mutations in the promoter region of SRBI, there was an increase in plasma cholesterol levels of 50–70% and an increase in size and cholesterol content of the HDL particles in mutant mice as compared to wild type. There was also a decrease in the hepatic uptake of free cholesterol (40%) and selective uptake of HDL cholesterol (50%) by mutant animals as compared to wild type [97]. Liver over-expression of SRBI in mice lead to 92–94% decrease in total plasma cholesterol levels as compared to wild type animals. There was also a decrease in plasma phospholipids (75%) and triglycerides (45–58%) levels as compared to wild type animals [98].

SRBI and its ligand HDL are of significance in the early steps of HCV infection. The identification of SRBI as a HCV cellular receptor was made with HCVpp *in vitro*. The infection of 293T cells by HCVpp was enhanced 10 fold with the over-expression of SRBI, and SRBI anti-serum reduced HCVpp infectivity of Huh7 and CD81^+ ^HepG2 cells in a dose dependent manner [49]. Infection of Huh7 cells by HCVpp was enhanced in the presence of HDL. The increase in infectivity was 5 fold if the HDL was added to the media after HCVpp binding to cells whereas the increase was only 1.7 fold when both were added simultaneously. This points to the possibility that HDL enhancement of HCVpp infectivity occurs post binding [50]. The production of HCVpp particles in presence of HDL or human serum increased infectivity of the produced virus in a dose dependent manner. There was not a significant increase in infectivity when the virus was grown in the presence of LDL or VLDL. The enhancement in infectivity was lost when cells were treated with anti-SRBI prior to infection or SRBI expression was attenuated. Enhancement was also lost, in a dose dependent manner, upon treatment of cells with drugs which block SRBI ability to uptake cholesterol esters from HDL [51].

As with the HCVpp, similar results are seen with the HCVcc *in vitro *system. Infectivity of HCVcc, grown in serum free media, increased up to 2 folds with introduction of HDL, but decreased with increasing concentrations of HDL. At HDL levels equivalent to physiological concentrations, HDL was inhibitory for HCVcc infection of Huh7 cells [99]. In a separate study, HCVcc infectivity of Huh7.5 cells over-expressing SRBI increased 18 fold as compared to parental cells. The over-expression of SRBI in Huh7.5 cells led to an increase in cell to cell spread and secondary infections by HCVcc [54].

The important roles of SRBI and HDL in HCV infection has led to a closer look at the effects cholesterol has on infectivity of HCV. The depletion of cholesterol, by 60%, from Huh7 cells prior to infection with HCVcc resulted in a 6.2 fold inhibition of infectivity. Inhibition was reversed upon treatment of cells with exogenous cholesterol [55]. The cholesterol/phospholipid ratio of HCVcc was found to be 1.29, as compared to a ratio of 0.4 and 0.42 for cell membranes of non-infected and infected cells, respectively. A decrease of HCVcc cholesterol levels led to a decrease in the infectivity of the virus [100]. These results indicate the importance in cholesterol levels to infectivity which further highlight the role SRBI plays directly and in-directly in HCV infection.

The effective interaction of the HCV glycoproteins, SRBI, and CD81 are necessary for a productive infection to occur. Experimental results have shown complex formation between HCV E2, CD81, and SRBI. Removal of one protein abrogated formation of any complex between the remaining proteins [78]. In the case of HCVcc infection, synergistic inhibition of infectivity was observed when cells were pretreated with both anti-CD81 and anti-SRBI, as compared to treatment with one antibody. The authors concluded their results point to CD81 and SRBI functioning cooperatively during the infection process. Although both CD81 and SRBI are needed for a productive HCVpp infection, there was a lack of synergy when blocking both receptors which points to a lack of cooperativity between the two receptors in a HCVpp infection [55].

### Claudin-1

Claudins are transmembrane proteins involved in the formation of tight junctions. Their tetraspan transmembrane topology produces two extracellular loops, one intracellular loop, and two intracellular tails (the C and N-terminus). Within the family of mammalian claudins the N-terminal is ~7 amino acids, the first extracellular loop (ECL1) is ~50 amino acids, the intracellular loop ~12 amino acids, the second intracellular loop (ECL2) is ~25 amino acids, and the C-terminal 25–50 amino acids [101-103].

A general function of claudins, in tight junction formation, is paracellular sealing. Claudin-1, -5, -11, and -14 knock out mice have shed light on the function of these proteins, *in vivo*, in the tightening of skin [104], the blood brain barrier [105], myelin sheets and Sertoli cell layers [106], and the epithelial in the inner ear [107], respectively. The distinct properties of a given tissue and its relationship to its tight junctions seem to be largely dependent on the combination of claudins that are expressed and on the manner they copolymerize [103,108,109]. Claudin-1 is highly expressed in the liver and is also found in other epithelial tissues [110].

Performing a cyclic lentivirus based repackaging screen of a complementary DNA library, derived from a highly permissive cell line to HCV infection, for genes that confer susceptibility to HCV infection to non-permissive cell lines, claudin-1 was identified as a cellular receptor for HCV [111]. Claudin-1 is expressed in all hepatoma cell lines permissive to HCVcc and HCVpp infection, except for Bel7402 [112], as well as primary hepatocytes [113]. The expression of claudin-1 in 293T cells enhanced HCVpp infection, in one study by more than a 100 fold [111] and another to the same levels as HCVpp infection of Huh7.5 cells [113]. HCVpp infection of 293T cells expressing claudin-1 was inhibited by serum from HCV^+ ^patients, anti-CD81, and bafilomycin A1, demonstrating that HCVpp entry was also dependent on the envelope glycoproteins, CD81, and endosomal acidification [113]. Claudin-1 expressing 293T cells were also permissive to HCVcc infection, although efficiency of infection was 1000 folds less than Huh7 cells [111]. The overexpression of claudin-1 in cell lines permissive to HCVpp infection did not enhance infectivity [111].

HCVpp infection remained CD81 dependent even when claudin-1 was overexpressed in Hep-G2 (CD81 negative cell line, becomes susceptible to HCVpp upon expression of CD81) cell line. The expression of murine claudin-1, instead of human claudin-1, did not negatively effect HCVpp susceptibility which suggests that claudin-1 in not a determinant of specie host range of the virus. Down regulation of claudin-1 via siRNA resulted in a decrease in infection levels of HCVpp and HCVcc [111].

The n-terminal 1/3 of extracellular loop 1 (ECL1) was identified as sufficient for HCVpp entry when expressed in a claudin-7 background. Of the 5 residues that differ between the claudin-1 and claudin-7 in the n-terminal 1/3 of ECL1, 2 were found to be important in regard to HCVpp infection. The introduction of M32I or K48E into claudin-7 rendered 293T cells partially permissive to HCVpp infection, but the combination of both mutations supported HCVpp entry as efficiently as claudin-1 [111].

Post binding antibody inhibition of claudin-1 demonstrate that, like CD81 [62], claudin-1 acts at a post-binding stage in HCV infection. The results of these experiments suggest a sequence in which CD81 interacts with the virus prior to claudin-1 [111]. Cell to cell fusion studies also demonstrated that claudin-1 is required for HCV envelope glycoprotein mediated fusion although it is unclear if claudin-1 participates directly in the fusion process or that its involvement is required in an earlier step [111].

Two other family members of claudin-1, claudin-6 and -9, have been identified as possible HCV cellular receptors. The expression of claudin-6 and -9 in 293T cells resulted in the cells becoming permissive to HCVpp infection at similar levels as Huh7.5 [112,113] and permissive to HCVcc infection, but at titers 400 times lower than those achieved in Huh7.5 cells [112]. Interestingly, the attenuation of claudin-1 expression and expression of either claudin-6 or -9 in Huh7.5 cells, lead to abrogation of HCVcc permissiveness. Furthermore the expression of claudin-6 and -9 in claudin-1 negative hepatoma cell lines was not effective in conferring the ability to become HCVpp permissive, but the expression of claudin-1 made the cell line permissive to HCVpp infection [113]. The exception seems to be the HCVpp permissive hepatoma cell line Bel7402, which is claudin-1 negative but expresses claudin-9. The reduction in claudin-9 expression in Bel7402 led to the a significant decrease in HCVpp infectivity [112].

### LDLR

The LDL receptor (LDLR) is a single pass transmembrane glycoprotein of 839 amino acids. It is a modular proteins consisting of seven adjacent LDL receptor type-A (LA) modules at the n terminal end, followed by a region of homology to the epidermal growth factor precursor (EGFP) which consists of two epidermal growth factor-like (EGF) modules, a YWTD domain, a third EGF module, a serine and threonine rich region, a transmembrane region, and a 50 residue cytoplasmic tail [114-116].

The LDLR receptor is responsible for the cellular sequestering of cholesterol containing LDL and VLDL particles from circulation. The underlying genetic cause familial hypercholesterolemia (FH) is a loss of function mutations in the LDLR gene. FH is an autosomal genetic disorder affecting approximately 1 in 500 individuals worldwide. In heterozygous individuals, FH presents as an increased risk of atherosclerosis and coronary heart disease. Homozygous individuals, if untreated, typically die of heart disease at an early age [116]. The LA repeats have been shown to be the ligand binding domain of LDLR [117].

A majority of plasma cholesterol in humans circulates in the form of LDL. LDL is the primary ligand for the LDLR and consists of one copy of apolipoprotein B-100 (apoB-100) as its primary protein component. The LDLR receptor also binds, with high affinity, lipoproteins which contain multiple copies of apolipoprotein E (apoE), such as the β-migrating forms of very low density lipoprotein (β-VLDL) and some intermediate density lipoproteins [118,119]. LDLR binding of apoE requires apoE association with lipids [120]. LDLR-ligand complexes enter cells via clathrin-coated pits and are then delivered to endosomes where the low pH environment triggers the release of ligand from receptor. The receptor is then returned to the plasma membrane in a process called receptor recycling. The lipoprotein particle proceeds to the lysosome where the hydrolysis of the released cholesterol esters occurs [121].

There is evidence that LDLR is involved in the HCV infection process. The binding of HCV particles, from HCV positive serum of patients, to human dermal fibroblasts were inhibited by pretreatment of cells with >200 μg/ml of purified LDL. The expression of LDLR on COS-7 cells led to HCV binding to the cells from 7 out of 12 patient sera [25]. Further evidence for the role of LDLR in HCV infection was gathered by studies done with primary human hepatocytes. A peptide inhibitor of LDL binding to LDLR inhibited HCV infection of hepatocytes. This effect was most potent when the peptide was added at the time of infection and the inhibitory effect diminished progressively when peptide was added at time points after infection. This results suggests that LDLR is involved in viral attachment to the hepatocytes. Treatment of hepatocytes with monoclonal antibodies against LDLR or LDL also inhibited HCV infection [26]. These findings and the association of HCV particles with lipoproteins suggest a role for LDLR as a cellular receptor for HCV.

### Glycosaminoglycans

Glycosaminoglycan (GAG) chains on cell surface proteoglycans serve as attachment sites for the binding of a number of viruses and other microorganisms. GAG chains are ubiquitously present on the cell surface of eukaryotic cells with varying composition and concentration dependent on cell type [122]. The GAG heparan sulfate comprises of a family of linear polysaccharides with a signature motif of repeating units of [GlcA-GlcNAc]_n_, where GlcA is glucuronic acid and GlcNAc in *N*-acetylglucosamine. The saccharides undergo N deacetylation and N sulfation of the GlcNAc residues, O sulfation at other positions, and epimerization of GlcA to iduronic acid, which gives rise to structural diversity throughout the length of each chain [123].

The GAG heparan sulfate has been identified as a HCV cellular receptor [20,22-24,62]. Heparin, a close structural homologue of highly sulfated heparan sulfate, was able to bind HCV E2 in an ELISA, in a concentration-dependent manner. The dissociation constant, K_d_, for E2 and E1 binding to heparin was measured at 5.2 × 10^-9 ^M and 5.3 × 10^-8 ^M, respectively [20,22]. The binding of E2 to HepG2 cells was inhibited in a dose-dependent manner by pre-incubation of E2 with heparin and liver derived highly sulfated heparan sulfate [20].

The pretreatment of HCVpp with heparin and highly sulfated heparan sulfate led to marked inhibition in infectivity of Huh7 cells with an IC_50 _0.5 μg/ml. If HCVpp was allowed to bind Huh7 cells prior to the addition of heparin or highly sulfated heparan sulfate, the inhibitory effect was not as dramatic [22,23]. The pretreatment of HCVcc particles with heparin led to a dose dependent inhibition of HCVcc binding at 4°C to Huh7 cells, and the pretreatment of Huh7 cells with heparinase II and heparinase III inhibited HCVcc binding to Huh7 cells at 4°C by 51–75% and 60–75%, respectively [24].

The incubation of HCVcc particles with heparin led to a moderate dose dependent inhibition of HCVcc infection of Huh7 cells with an IC_50 _value of 50 μg/ml. This inhibitory effect was not observed if Huh7 cells were pre-treated with heparin prior to the addition of virus implying direct interaction of HCVcc with heparin is responsible for the inhibition observed. The pretreatment of Huh7 cells with heparinase I and III also led to a moderate inhibition of HCVcc infectivity (40–60%). Heparin's inhibitory effect on HCVcc infection of Huh7 cells was abrogated if administered to cells after viral binding had taken place [62]. This, and other findings, indicate that cellular GAG, and specifically highly sulfated heparan sulfates, are involved in the process of HCV binding to cells.

### Occludin

A recent study has identified occludin (OCLN) as a HCV cellular receptor [124]. OCLN is a four transmembrane domain protein present in the tight junctions of polarized epithelial cells. HCV permissive human hepatoma cell lines such as Huh7 or cell lines shown to lack other entry factors (i.e. HepG2 and 293T cells) were found to express detectable levels of OCLN. Overexpression of OCLN did not enhance susceptibility to HCVpp infection. Silencing of OCLN expression lead to inhibition of HCVpp infection in Hep3B cells and inhibition of infection of both HCVpp and HCVcc in Huh-7.5 cells. These observations indicate that OCLN is essential for HCV infection of naturally permissive cell lines. Overexpression of human OCLN in HCV resistant cell lines, which express the other entry co-receptors, led specific enhancement in susceptibility to HCVpp infection.

### Liver tissue expression of HCV receptors

The expression levels and localization of the known HCV receptors in normal and infected liver was examined and published by Dr. McKeating's laboratory [125]. In a normal liver, CD81 expression on hepatocytes was observed on the basolateral surface with some canalicular expression. CD81 expression was also present in the stroma of the portal tracts. SRBI expression was seen on the sinusoidal endothelium and hepatocytes. There was minimal amount of SRBI expression observed on the bile ducts and hepatocyte expression was located at the basolateral surface. Claudin 1 expression was seen on the bile ducts and hepatocytes, with low levels of expression on the sinusoidal endothelium. Hepatocyte expression of claudin 1 was observed on the basolateral and canalicular membranes. In a HCV infected liver an increase in claudin 1 expression was observed on the basolateral membrane of hepatocytes. In normal liver tissue, the co-localization of claudin 1 and CD81 was observed to be the strongest in the apical-canalicular region. In HCV infected liver tissue, the co-localization was prominently observed at the basolateral region. Claudin 1 and SRBI co-localization was seen at the basolateral membrane in both normal and HCV infected liver tissue.

## Conclusion

This review summarizes the role each HCV cellular co-receptor in the infection process and the endogenous function of each of these co-receptors. Much has been learned in the past few years of the mechanism and requirements for HCV to successfully infect naïve cells. With future advances in developing robust *in vivo *(i.e. small animal model of HCV infection) and *in vitro *(i.e. infection of primary hepatocytes, HCVcc strains of different genotypes) assays our understanding of the processes involved in the early steps of HCV infection will be greatly expanded.

## Competing interests

The author declares that they have no competing interests.

## Authors' contributions

AS is the sole author of this manuscript.

## Author information

After finishing high school in Tucson, Arizona, the author enlisted as an infantryman in the United States Army. After three years of military service, he attended Southern University in Baton Rouge, Louisiana, where he earned a bachelor of science degree in physics. He then enrolled for 2 years as a graduate student at the chemistry department at Tulane University after which he joined the Molecular and Cellular Biology Program at Tulane University Medical Center and the joined the laboratory of Dr. Robert F. Garry in 2003 and began his work on the hepatitis C virus. The author successfully defended his dissertation in December of 2008.
